# Three-Dimensional Rotating Wall Vessel-Derived Cell Culture Models for Studying Virus-Host Interactions

**DOI:** 10.3390/v8110304

**Published:** 2016-11-09

**Authors:** Jameson K. Gardner, Melissa M. Herbst-Kralovetz

**Affiliations:** Department of Basic Medical Sciences, College of Medicine—Phoenix, University of Arizona, Phoenix, AZ 85004, USA; jamesongardner@email.arizona.edu

**Keywords:** bioreactor, emerging viruses, host immune mechanisms, human tissue engineering, in vitro cell culture, infectious disease, organotypic, pathophysiology, low fluid-shear, viral pathogenesis

## Abstract

The key to better understanding complex virus-host interactions is the utilization of robust three-dimensional (3D) human cell cultures that effectively recapitulate native tissue architecture and model the microenvironment. A lack of physiologically-relevant animal models for many viruses has limited the elucidation of factors that influence viral pathogenesis and of complex host immune mechanisms. Conventional monolayer cell cultures may support viral infection, but are unable to form the tissue structures and complex microenvironments that mimic host physiology and, therefore, limiting their translational utility. The rotating wall vessel (RWV) bioreactor was designed by the National Aeronautics and Space Administration (NASA) to model microgravity and was later found to more accurately reproduce features of human tissue in vivo. Cells grown in RWV bioreactors develop in a low fluid-shear environment, which enables cells to form complex 3D tissue-like aggregates. A wide variety of human tissues (from neuronal to vaginal tissue) have been grown in RWV bioreactors and have been shown to support productive viral infection and physiological meaningful host responses. The in vivo-like characteristics and cellular features of the human 3D RWV-derived aggregates make them ideal model systems to effectively recapitulate pathophysiology and host responses necessary to conduct rigorous basic science, preclinical and translational studies.

## 1. Introduction

In vitro studies of complex virus-host interactions require robust cell culture models that effectively recapitulate in vivo properties and characteristics. Researchers have utilized conventional two-dimensional (2D) monolayer cell cultures for many decades, increasing understanding in viral life cycles and the host immune response. Cells grown in conventional monolayer cell cultures, however, often lack polarization and the architectural features of in vivo tissues and, therefore, may improperly represent key virus-host interactions [1,2,3]. Additionally, many newly-emerging and difficult-to-propagate viruses (Zika virus, severe acute respiratory syndrome coronavirus and hepatitis viruses, for example) lack sufficient animal models and/or in vitro cell culture models to allow for their study. Therefore, there is a clear need for in vitro models that display key cellular components and features to accurately model virus-host interactions. The development of new therapeutic agents and treatments for viral infections requires a more complex understanding of virus-host interactions, as well as, culture systems that model the in vivo environment as accurately as possible. Viruses often require distinct cellular architectural features and polarized orientation with receptors for attachment and entry, which may not be present on cells grown in conventional monolayer cell cultures. For example, a recent review article highlighted the important role of tight junctions in viral entry, replication, dissemination and egress in at least nine different DNA and RNA viruses [4]. Tight junction proteins are also key components of the epithelial barrier function and integrity that protect against viral infection and potentially influence the susceptibility of tissue to viral infection. Mucin production at mucosal epithelial sites additionally influences virus-host interactions at the epithelial barrier and ultimately impacts viral infection and transmission [5,6,7]. While some of these features may be present in conventional monolayer cell cultures, they often lack the polarity and other topographic features that are present in vivo. Rotating wall vessel (RWV) bioreactors effectively model many of these features, including tight junctions and unidirectional expression of mucin and key receptor proteins, allowing investigators to more effectively study virus-host interactions as they exist in vivo in a reproducible fashion.

Early attempts at three-dimensional (3D) cell culture utilized explant tissue cultures [8,9]. The collection of explant tissue, however, is limited by donor availability, and such explants have a short lifespan in culture [10]. More recent attempts at 3D modeling have included scaffold-based systems, scaffold-free systems, transwells and microfluidics [11,12,13]. The National Aeronautics and Space Administration (NASA) initially developed the RWV bioreactor as a way to model the microgravity environment encountered in space and to investigate growth, regulatory and structural processes [14,15]. The RWV bioreactor was found to create a modeled microgravity, low fluid-shear environment that provides the necessary oxygenation and nutrients for development and polarization. In this environment, cells were observed to form cellular structures and features not readily expressed in conventional monolayer cell culture. Since its development, the RWV bioreactor has been utilized for the study of cellular and microbial gene expression in microgravity, cellular differentiation, host-pathogen interactions and tissue engineering [16,17,18,19].

**Figure 1:**
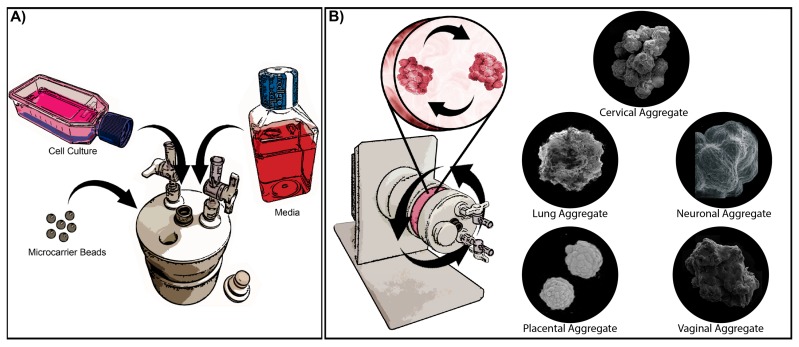
Culturing three-dimensional (3D) aggregates in the rotating wall vessel (RWV) bioreactor. (**A**) Cells are grown to confluence in two-dimensional (2D) conventional monolayer cell cultures, then are combined with microcarrier beads and appropriate media in the RWV bioreactor. Cells attach to the microcarrier beads in the bioreactor, and culture media can be replaced at any time according to the metabolic needs of the developing aggregates, as described in Radtke et al. [27]. (**B**) The RWV bioreactor is kept in constant rotation at a low speed to create a low fluid-shear simulated microgravity environment that prevents cell detachment and sedimentation. Attached cells grow and form cell-cell junctions creating large aggregates consisting of multiple microcarrier beads. Cells also polarize as they develop and express many characteristics of the parental tissue. Inserts at the right show scanning electron micrographs (SEM) depicting representative 3D aggregates of cells representing cervical, lung, neuronal, placental and vaginal tissues. The cervical tissue SEM is modified from Radtke et al. [28] with permission. The lung tissue SEM is taken from NASA/TP-2012-217363, Paramyxovirus infection mimics of in vivo cellular dynamics in 3D human broncho-epithelial tissue-like assemblies, and used with permission from NASA [30]. The neuronal tissue SEM is modified from Goodwin et al. [31] with permission. The placental tissue SEM is modified from McConkey et al. [26] with permission. This work is licensed under CC BY-NC (http://creativecommons.org/licenses/by-nc/4.0/). The vaginal tissue SEM is modified from Hjelm et al. [25] with permission.

In the RWV bioreactor, cells are cultured with microcarrier beads or matrices that allow the cells to attach and spontaneously develop 3D ultrastructures representative of the parental tissue. As the cells grow and develop, cellular aggregates form via bead-to-bead bridges, and cellular aggregates can be sampled at various time points to monitor the progress of development [20]. The low fluid-shear environment prevents the cells from detaching from the microcarriers and protects growing tissue aggregates from damage that can occur from excess agitation during culturing [20]. The low fluid-shear environment also promotes the co-localization of particles in the fluid and leads to the formation of the bead-to-bead bridges and cellular aggregates [15]. In addition, this low fluid-shear environment mimics the flow in vivo during development and thereby promotes cellular differentiation as cells signal and grow in 3D. Developing 3D aggregates are kept in a continuous free-fall state of neutral buoyancy that precludes aggregate sedimentation, while maintaining the low fluid-shear environment, and allows the cells to grow around the microcarrier and develop complex structures observed in vivo (Figure 1) [15,20,21,22,23]. In the RWV bioreactor, oxygenation occurs by the diffusion of dissolved gasses, creating a zero headspace environment that provides the necessary oxygenation for the developing aggregates while maintaining the low fluid-shear environment [22]. Collectively, these growth conditions allow for 3D tissue aggregates to develop and form organotypic ultrastructures that are not readily present in conventional monolayer cell culture and are necessary for advancing the development of in vitro models that effectively recapitulate in vivo tissue. For example, epithelial cells grown in the bioreactor express adhesion proteins, form desmosomes and tight junctions, produce secretory material and mucus and also form microvilli and microridges [14,24,25,26,27,28,29]. Notably, cells that are seeded into the bioreactor reflect their phenotype. For example, vaginal epithelial cells form a multi-layered stratified squamous epithelium, whereas endocervical epithelial cells form a single layer as found in vivo, therefore reflecting the authentic microanatomical features of the parental tissue [25,28]. Cancerous cell lines, on the other hand (e.g., MCF-7 cells), no longer require the extracellular matrix for growth, do not attach and grow on the collagen-coated microcarrier beads, thereby reflecting their cancer phenotype. Fully-differentiated aggregates can remain in the bioreactor for infection or aggregates can be harvested and plated for downstream experiments, including infections.

To date, a wide variety of cell lines and tissues has been successfully cultured and reproduced in the RWV bioreactor. The purpose of this review is to highlight those RWV bioreactor studies that focus on modeling virus-host interactions in various tissue settings (Table 1). Additionally, we discuss methodological advancements and future applications of these reproducible and robust RWV bioreactor-derived models for the continued study of virus-host interactions and maximizing their translational utility.

## 2. Virus Infection and Replication in 3D Bioreactor Models

Barrier features, including tight junctions, mucus secretion and microvilli, are not often present in cells grown in a conventional monolayer cell culture and influence virus-host interactions [4,46]. Tight junctional proteins are receptors for several viruses, like hepatitis C virus and adenovirus, and their expression in 3D cell culture can enhance viral attachment and entry [4]. The presence of tight junction proteins, mucus and other barrier features can also increase resistance to viral infections and more accurately represent in vivo virus-host interactions. Our studies, along with those from other researchers, have found that cells grown in the RWV bioreactor are more resistant to infection, requiring a higher virus multiplicity of infection (MOI) to productively infect 3D aggregates when compared to conventional monolayer cell culture [27,47,48]. Likewise, cells grown in the RWV bioreactor show increased resistance to bacterial infection, and infected 3D cell cultures display decreased bacterial replication when compared to infections in conventional monolayer cell culture [27,49,50,51]. A study of four veterinary viruses in 3D RWV bioreactor-derived aggregates further supported these findings by directly comparing the replication of the viruses in conventional monolayer cell cultures vs. 3D aggregates. Three-dimensional monkey kidney epithelial cell (VERO) aggregates and 3D bovine kidney epithelial cell (MDBK) aggregates were infected with two DNA viruses (Suid herpesvirus 1 and Bovine adenovirus) and two RNA viruses (Vesicular stomatitis virus and Bovine parainfluenza virus). Infections with all four viruses in the 3D aggregates produced a lower viral titer over the course of infection compared to conventional monolayer cell cultures [52]. However, despite being more resistant to infection, more infectious virions were produced in 3D aggregates, while the conventional monolayer cell culture produced more noninfectious virions [52]. These findings allow the speculation that the increased resistance to infection is due to the barrier features that polarized 3D aggregates exhibit when grown in the RWV bioreactor. The increased infectivity of the virions produced is also noteworthy and suggests again that the 3D aggregates better mimic the in vivo environment. For these and other reasons, we argue herein that the 3D cellular aggregates grown in the RWV bioreactor provide a more in vivo-like simulation of parental tissues, and their susceptibility to virus entry, replication and subsequent pathogenesis support their utilization for the study of these processes in vitro.

## 3. Three-Dimensional Models of Human Neuronal Cells to Study Persistent Viral Infections

Varicella zoster virus (VZV) is an alphaherpesvirus that belongs to the *Herpesviridae* family that establishes a latent infection in ganglionic neurons following an initial phase of acute infection. The virus will often reactivate in later years, causing zoster, a localized dermatomal rash, and also neurologic diseases, including meningoencephalitis and myelopathy. Over 90% of the worldwide population is seropositive, and although a vaccine has been developed and is currently available, there remains a risk of virus reactivation [53,54]. A lack of animal models and limited availability of VZV-free human ganglionic neurons have limited the study of the virus. Recently, normal human neuronal progenitor (NHNP) cells have been utilized to study VZV infection and latency. When NHNP cells are cultured in the RWV bioreactor, they become partially differentiated, leading to the formation of 3D aggregates that display features observed in human trigeminal ganglia [31]. These 3D aggregates express mature neuronal markers, such as glial fibrillary acidic protein, neuron-specific nuclear protein, β-tubulin III and microtubule-associated protein A and B after 180 days in culture [31]. Three-dimensional NHNP aggregates also express additional neuronal markers (nestin and tubulin) at levels similar to those seen in human trigeminal ganglia; however, late stage neuronal development markers CD105, CD90 and CD49f are expressed at lower levels [31].

Three-dimensional NHNP aggregates support persistent VZV infections with limited or little lytic replication and sporadic reactivation at later time points [31]. NHNP cellular aggregates infected with fluorescently-labeled VZV in the bioreactor showed a significant increase of VZV genome copies over an 18-day period, yet aggregates remained viable in culture over a three-month course of infection [31]. The VZV genome was able to stably replicate, and infectious virus progeny was detected in the cell culture supernatant intermittently throughout the course of infection [31]. The maintenance of viable 3D aggregates over a three-month course of infection, while not exactly modeling in vivo infections, does allow for the study of persistent VZV infections over an extended period of time. Prolonged studies of VZV infections enable researchers to identify key interactions that may influence virus gene expression, replication and potentially establishment of latency. Prior to the development of the 3D NHNP aggregates, many other cell lines had been utilized to study VZV infection, including human neuroblastoma (IMR-32), monkey kidney epithelial (VERO), primary human foreskin fibroblasts (HFF), human melanoma (MeWo) and peripheral blood mononuclear cells (PBMC). In these cell lines, however, VZV infections are lytic and preclude prolonged culturing and the study of VZV latency and reactivation cycles [31,55,56,57,58]. It should be noted that a non-lytic VZV infection was achieved with differentiated human neural stem cells (NSC); however, these infections were nonproductive [59]. Despite its limitations, the development of 3D NHNP aggregates grown in the RWV bioreactor represents a step forward in the study of VZV virus-host interactions. Further development of 3D aggregates to model virus latency and reactivation can provide new insights into the mechanisms of VZV pathogenesis and could potentially be utilized in the study of other neurotropic viruses, such as other herpesviruses.

## 4. Three-Dimensional Models of Lymphoid Tissue and Circulating Lymphocytes for Long-Term Culture to Study Virus Replication, Latency and Reactivation

One of the first viruses studied utilizing the RWV bioreactor developed by NASA was Epstein–Barr virus (EBV) (Table 1). EBV is a member of the *Herpesviridae* family, and approximately 90% of adults are seropositive for the virus [60]. The virus infects epithelial cells and B lymphocytes and establishes latency in resting memory B cells [61]. In humans, B cells circulate through the periphery, often in a quasi-gravity state where gene expression and cell metabolism may be different from that observed in conventional monolayer cell culture [33]. Simulated microgravity has been shown to alter the gene expression, proliferation and cellular interactions of non-adherent cells compared to when they are cultured in non-rotating, static environments [62,63]. As already noted, the RWV bioreactor provides a simulated microgravity environment to reproduce conditions in circulation and is therefore ideally suited for the in vitro study of EBV-infected B cells. P3HR-1, Daudi and Ramos B-cell lines have been cultured in the RWV bioreactor to study factors influencing EBV latency and reactivation [33,34,35]. Indirect immunofluorescence assays showed that EBV-positive P3HR-1 and Daudi cells cultured in RWV bioreactors displayed significantly lower expression levels of lytic cycle proteins compared to cells grown in conventional monolayer cell culture, suggesting that EBV reactivation is suppressed in microgravity environments [33].

Human immunodeficiency virus (HIV) is a member of the *Retroviridae* family, which also infects lymphoid tissue and circulating lymphocytes. In addition to the formation of 3D aggregates from cells grown in conventional monolayer cultures, the RWV bioreactor can be utilized for culturing tissue blocks in the simulated microgravity environment, thereby maintaining their in vivo cellular organization and structure while allowing for the delivery of nutrients [32]. Margolis et al. inserted blocks of human tonsil tissue into the RWV bioreactor and cultured these blocks with additional cells from the same tonsil. These cultures were not only viable for up to three weeks, but also contained lymphocytes that migrated throughout the tissue and cell culture media. Infection of these cultures with HIV-1 isolates was productive, with exponential viral replication during the first week of infection [32]. In situ hybridization for HIV RNA confirmed a productive infection, with 1%–3% of the cells in the tissue blocks becoming infected [32]. Flow cytometry demonstrated a decrease in CD4^+^ cells over the course of the infection, confirming the productive infection of CD4^+^ T cells [32]. Additionally, transfer of HIV-positive tissue blocks to RWV bioreactors containing HIV-negative tissue blocks led to the infection of the HIV-negative tissue blocks, and virus was detected in the cell culture supernatant [32]. Culturing tissue blocks in the RWV bioreactor thus creates an in vitro model system that could provide new insights into HIV infections and virus interactions with lymphoid tissue.

## 5. Three-Dimensional Models for Respiratory Viral Infections

The respiratory epithelium forms a robust barrier to prevent infection. Tight junctions, cilia and mucus secretion are some of the important features of this barrier. Conventional cell culture techniques are often used for the study of respiratory viruses, but lack many of the important features of the intact human respiratory epithelium, like mucus, microvilli and cell-cell junction proteins. Cultures of 3D respiratory epithelial cell aggregates have therefore been applied to the study of the host immune response to bacterial infections in the lungs and can be effective models for studying emerging respiratory viruses and difficult-to-propagate viruses that previously lacked a robust model for study [49].

Two widely-prevalent paramyxoviruses, parainfluenza virus type 3 (PIV3) and respiratory syncytial virus (RSV), cause severe respiratory disease in young children. Both of these viruses have proven to be difficult to propagate in conventional cultures and also lack robust animal models that accurately model viral infection, pathogenesis and the host immune response [64]. Recently, it has been shown that immortalized human bronchotracheal epithelial (BEAS-2B) cells and primary normal human bronchial epithelial (NHBE) cells grown as 3D aggregates in the RWV bioreactor are permissive to RSV and PIV3 infection (Table 1). Transmission electron microscopy further demonstrated that the aggregates grow in a multi-layered structure that exhibits tight junctions and microvilli, thus displaying key features of the in vivo tissue [37]. These respiratory 3D aggregates have been cultured for more than 35 days while maintaining functional cell markers and, when challenged with RSV, display cellular damage and active viral infections, including budding virions [37]. Cytokine profiles from the RSV-infected and PIV3-infected 3D lung epithelial aggregates demonstrated an induction of pro-inflammatory cytokines, chemokines and other immune factors including interleukin (IL)-1β, IL-8, macrophage inflammatory protein 1 alpha (MIP-1α), regulated on activation, normal T cell expressed and secreted (RANTES) and granulocyte-colony stimulating factor (G-CSF) at levels that were similar to those observed in nasal washes from children with RSV and PIV3 infections [30]. In contrast, cytokine profiles from conventional monolayers infected with RSV and PIV3 were not similar to the levels observed clinically. Taken together, these data support the utilization of 3D aggregates as models of in vivo RSV and PIV3 infections with regard to the function of epithelial cells in host response and barrier defense to these viruses [30].

Severe acute respiratory syndrome coronavirus (SARS-CoV) is an emerging virus first identified in China in 2002 [65]. Animal models for SARS-CoV have been developed, although there currently are no Food and Drug Administration (FDA)-approved vaccines or antivirals available [66]. Initial experiments with SARS-CoV suggest that 3D human bronchotracheal epithelial (BEAS-2B) aggregates provide a more physiologically-relevant and species-specific model that may be more permissive to viral infection by SARS-CoV and potentially other emerging human viruses (Table 1). Characterization of the 3D aggregates has revealed cellular differentiation and increased expression of collagen IV, mucin 1 (MUC1) and tight junction protein zonula occludens-1 (ZO-1) at levels similar to in vivo human tissue [36]. Infectious virions were not detected in cell culture supernatants by the standard plaque assay when the 3D aggregates were infected with SARS-CoV; however, immunocytochemistry illustrated cross-reactivity with antibodies to viral spike and nuclear proteins at the cytoplasm and plasma membrane in SARS-CoV-infected aggregates [36]. Cytoplasmic vacuoles also increased over the course of the infection; mitochondria became swollen and decreased in number; and endoplasmic reticula were disrupted; all clear signs of viral infection [36]. At 10 days post infection (DPI), cells began shedding off the microcarrier beads, but again, no budding virions were observed at any time during the course of infection [36]. Nevertheless, the positive immunocytochemistry and the visualization of cytoplasmic vacuoles appear to confirm a permissive SARS-CoV infection of the 3D aggregates, suggesting that these cultures could provide useful information about SARS-CoV infection and pathogenesis, and contribute to the development of novel interventions even in the absence of virion formation. These initial studies with 3D respiratory aggregates are encouraging and could be applied to other newly-emerging respiratory viruses, such as Middle East respiratory syndrome coronavirus (MERS-CoV).

## 6. Three-Dimensional Models for Studying Viral Gastroenteritis

Over 200,000 children under the age of five die every year from human norovirus (HuNoV) infections, with norovirus infection representing the second leading cause of diarrheal death in this age group [67,68,69]. An effective cell culture model for modeling HuNoV infections and interactions with intestinal epithelial cells has been elusive, slowing the development of urgently-needed interventions for this deadly illness [70].

Early studies investigated over 27 cell lines and approximately 33 different HuNoV strains for the propagation of the virus using in vitro monolayers and were unsuccessful [71]. It was hypothesized that 3D structures or other co-factors may be required for successful cultivation of HuNoV in vitro. Human small intestine (INT-407) epithelial cells were initially grown in the RWV bioreactor with collagen-coated beads to form 3D aggregates to study HuNoV (Table 1) [40,41,43,44]. INT-407 epithelial aggregates have been shown to differentiate and subsequently stain positive for tight junction markers occludin, claudin-1, epithelial cadherin (E-cadherin) and ZO-1 by immunofluorescence [40]. The 3D aggregates possess apical microvilli, though these microvilli are shorter than those on in vivo human intestinal epithelial cells [43]. Three-dimensional aggregates developed from an alternate cell line, Caco-2 colon carcinoma, also express tight junction markers ZO-1 and occludin, and apical microvilli on these aggregates were more similar to the human intestinal epithelium in vivo [24,43,44]. The 3D Caco-2 aggregates also displayed increased expression of MUC1, MUC13 and MUC17, which are highly expressed mucins in the intestine that could potentially influence virus-host interactions [24]. Both 3D Caco-2 and INT-407 aggregates also expressed histo-blood group antigens (HBGA) H1 and H2, cellular receptors for HuNoVs [24,43,72]. INT-407 and Caco-2 aggregates were challenged with HuNoV and harvested for viral RNA titers as measured by reverse transcription PCR (RT-PCR). Straub et al. reported successful HuNoV infection and detected viral replication in 3D INT-407 and 3D Caco-2 aggregates; however, these results have not been able to be replicated in either 3D INT-407 or 3D Caco-2 intestinal epithelial models [40,41,42,43,44]. Additionally, the INT-407 cell line has been reported to be contaminated with HeLa cells, and therefore, could explain why these studies of HuNoV cannot be replicated, and is not a robust or reproducible model for the study of HuNoV-host interactions [73,74].

Recent advances in the culture of HuNoV demonstrated the need for additional factors, including gut bacteria and/or bile, for robust HuNoV replication. Bacteria from the gut have been shown to express HBGA and facilitate HuNoV infection of human B cells [75]. Additionally, it has been shown that intestinal milieu, including bile, is an important factor that enhances HuNoV replication in human intestinal enteroids [76].

Although 3D Caco-2 aggregates alone do not support productive HuNoV infection, they possess in vivo-like properties that can aid researchers in understanding the interactions between intestinal epithelial cells and other gastrointestinal viruses, like Coxsackie virus B (CVB). Currently there is no vaccine for CVB, and CVB infections cause mild gastroenteritis, while myocarditis and persistent infections have been linked to type 1 diabetes development [77,78]. Three-dimensional Caco-2 aggregates have supported productive CVB infection and produce more infectious virions than infections in conventional monolayer cell culture (Table 1) [24]. Further study of CVB and other enteric viruses in 3D colonic epithelial aggregates promises new insights into the factors influencing viral entry and host-virus interactions.

## 7. Three-Dimensional Models of Liver Tissue for Studying Hepatitis Viral Infections

Two hepatotropic viruses, hepatitis C virus (HCV) and hepatitis E virus (HEV) have proven difficult to propagate in vitro, and only in recent years have researchers been able to study these viruses in a conventional cell culture.

HEV is a member of the *Hepeviridae* family and causes acute liver disease in humans. Transmission of certain HEV genotypes can be zoonotic (from pigs) and affects much of the developing world, especially East and South Asia [79]. Human cell culture models for HEV include PLC/PRF/5 hepatocarcinoma cells grown in conventional monolayer cell culture, which have inconsistently supported viral replication [39]. PLC/PRF/5 have been grown in the RWV bioreactor, where they form aggregates that also support viral replication (Table 1) [39,80,81]. The 3D aggregates become fully differentiated after a 28-day culture period and are viable in culture for over five months [39]. HEV RNA has been detected in supernatants from RWV cultures at the majority of collection points over a 175-day period [39]. In contrast, PLC/PRF/5 cells grown in conventional monolayer cell cultures contain no HEV RNA in the supernatants [39]. Scanning electron microscope (SEM) micrographs of RWV bioreactor supernatants demonstrated virions, and subsequent experiments showed that these virions are infective, confirming a productive infection [39]. Taken together, these data support the use of the RWV bioreactor to study the virus-host interactions that influence HEV infectivity and transmission in a culture system that closely resembles in vivo liver tissue.

HCV is a member of the *Flaviviridae* family and has chronically infected over 185 million people worldwide [82]. Despite this massive prevalence, the lack of model systems for HCV has limited researchers’ ability to study virus-host interactions in vitro. Conventional monolayer cell cultures of Huh7 human hepatoma epithelial-like cells are permissive to HCV infection, but have decreased expression of cellular features, including occludin and claudin-1, which are known to impact viral uptake (Table 1) [38]. Three-dimensional Huh7 aggregates, in contrast, provide a more physiologically-relevant system that is highly permissive to HCV infection. Light micrographs have shown that fully-differentiated aggregates are multilayered, and RT-PCR of cellular RNA showed increased expression of hepatocyte nuclear factors that regulate hepatocyte differentiation [38]. In addition, aggregates stained positive for tight junction proteins (occludin-1 and E-cadherin), cell adhesion proteins and HCV receptors (CD81 and SR-B1) by immunofluorescence [38]. This degree of differentiation and polarization of Huh7 cells allows for the studies of the interactions between HCV and barrier proteins regulating viral entry that were previously impossible in conventional monolayer cell culture. Three-dimensional Huh7 liver aggregates have been productively infected with HCV, and viral RNA was detected in aggregates throughout a two-week period of infection [38]. Infection of the Huh7 aggregates was further confirmed through immunofluorescence at days 1, 7 and 14 post-infection [38]. Cellular differentiation of the 3D aggregates and the increased expression of tight junction proteins enables the study of these difficult-to-propagate viruses and could be utilized in the study of other hepatotropic viruses.

## 8. Three-Dimensional Models of Female Reproductive Tract Tissues for Studying Sexually-Transmitted Infections and Emerging Viruses

The female reproductive tract (FRT) is divided into the upper tract, consisting of the endocervix, uterus, placenta, fallopian tubes and ovaries, a transitional ectocervix zone and the lower tract, consisting of the vagina and vulva [83]. The FRT is exposed to a myriad of microbes, including sexually-transmitted pathogens, many of which are able to establish chronic infections that are difficult to clear and can cause long-term reproductive and gynecologic sequelae. However, site-specific differences in the epithelial structure between the upper and lower genital tract necessitate the need to model these different tissues for the study of sexually-transmitted infections (STI) and other viruses. Our laboratory was the first to develop and characterize 3D models of human cervical and vaginal tissue using the RWV bioreactor [25,27]. Human models of the upper FRT, including the endometrium, have also recently been developed and characterized in our laboratory (unpublished data). In addition, human placental models have been successfully created and used to model microbial resistance at this site [26,29,45,84].

**Figure 2:**
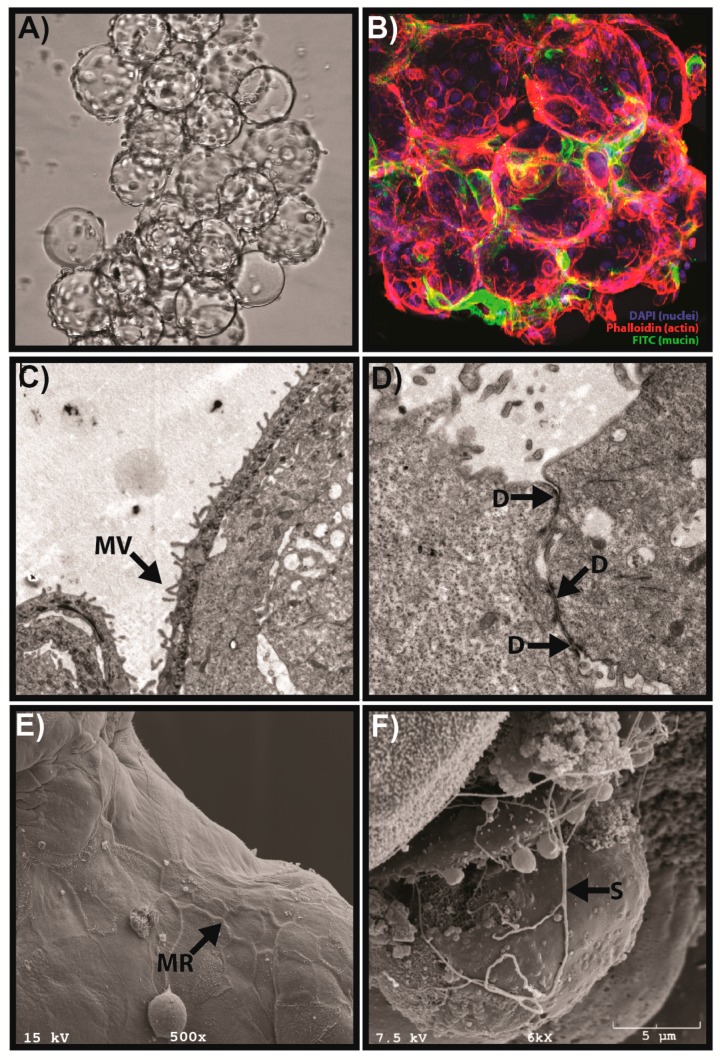
Example of physiological features of the RWV bioreactor-derived human 3D vaginal aggregates. (**A**) Phase micrograph of 3D vaginal epithelial cell aggregates consisting of multiple microcarrier beads connected by cell-cell junctions at an early stage of development. (**B**) Immunofluorescence of 3D aggregates by laser scanning confocal microscopy demonstrating localized mucus secretion modified from Hjelm et al. [25] with permission. Nuclei are stained with DAPI (blue); actin filaments are stained with phalloidin (red); and mucin 1 (MUC1) is stained with FITC (green). (**C**) Transmission electron microscope (TEM) image of 3D vaginal aggregates showing microvilli (MV), modified from Hjelm et al. [25] with permission. (**D**) TEM micrograph depicting desmosomes (D) at cell-cell junctions between vaginal epithelial cells. (**E**) SEM image showing formation of microridges (MR) at cell-cell junctions during late-stage development of 3D aggregates. (**F**) Production of secretory material (S) by vaginal 3D aggregates as shown by SEM.

Human vaginal epithelial (V19I) cells cultured in the RWV bioreactor develop in vivo-like properties that are not present when cells are grown in conventional monolayer cell cultures [25]. Scanning and transmission electron microscopy of 3D vaginal aggregates has demonstrated the presence of tight junctions/desmosomes, microridges, microvilli and mucus secretion, all of which influence viral interactions with host epithelial cells (Figure 2) [25,27]. The 3D aggregates can be used to test vaginal microbicides and recapitulated toxicity and host cytokine responses similar to human explant tissue, thereby demonstrating the translational capability of this model [25]. Three-dimensional cervical and vaginal aggregates exposed to microbial products, including poly(I:C) (a viral mimic and toll-like receptor 3 agonist), exhibit the induction of acute-phase and pro-inflammatory cytokines and mucus secretion, similar to primary human cells, and have been used to model the host immune response to viral infections (unpublished data) [27,85].

Herpes simplex virus type 2 (HSV-2) is the leading cause of genital herpes infections and causes persistent, life-long infections that increase the risk for STI and HIV acquisition [86]. Although antiviral drugs are available, viral reactivation and shedding can occur in many who are asymptomatic, and an effective vaccine remains elusive [87]. Vaginal and cervical epithelial aggregates are susceptible to FRT pathogens, including HSV-2, as shown by immunofluorescence and the standard VERO plaque assay (Table 1) (unpublished data) [27]. As mentioned previously, a higher MOI is required to infect 3D vaginal or cervical aggregates relative to conventional monolayers, most likely due to enhanced epithelial barrier features. HSV-2 infections of 3D vaginal aggregates induce secretion of cytokines, chemokines, mucus and anti-microbial products (AMP) and may provide a more accurate host immune response relative to conventional monolayers (unpublished data). More recently, our laboratory has reported that exposure of 3D human vaginal and cervical aggregates to microbial products, including poly(I:C), induces the expression of a novel pro-inflammatory cytokine, IL-36γ [85]. While this cytokine has been identified at other mucosal sites, this was the first report of this cytokine in the FRT and was validated with human tissue [85,88,89]. We hypothesize it may play a key role in mucosal host defense at this site. Collectively, these physiologically-relevant features support the use of 3D FRT aggregates from RWV bioreactors to study host immune mechanisms for other viral STI, including HIV and human papillomavirus.

The placenta is an important site for the exchange of nutrients and gasses during pregnancy, and virus-host interactions at the placenta have significant health implications for both mother and developing fetus. Human choriocarcinoma trophoblast cells (using the JEG-3 cell line) have been shown to produce 3D aggregates in the RWV bioreactor when the cells are co-cultured with placental microvascular cells [26]. The JEG-3/microvascular aggregates form over a 21-day period and display increased expression of placental differentiation markers, including human chorionic gonadotropin beta subunit (βhCG), human placental lactogen (hPL), syncytin, placental protein 13 (PP13) and major facilitator superfamily domain-containing protein 2 (MFSD2) [26]. Transcriptome profiles of the 3D aggregates were significantly different from the profiles of cells grown in conventional monolayer and were more similar to profiles from primary human trophoblasts [26]. The 3D aggregates also form syncytia and brush borders that are additional markers of cellular differentiation and that are not observed in conventional monolayer cell culture [26]. The syncytia and brush borders present in the 3D aggregates provide resistance to vesicular stomatitis virus (VSV) infection, whereas cells in conventional monolayers remain susceptible to VSV infection as shown by RT-PCR [26]. These results support the protective nature of placental trophoblasts (both in vivo and in 3D aggregates) against VSV infection. This 3D placenta model may also be useful for studying the interactions between emerging viruses, including the Zika virus and its severe effects in pregnancy (e.g., irreversible microcephaly) observed in newborn babies from Zika-infected mothers [90].

## 9. Future Directions for RWV Bioreactors

### 9.1. Considering the Site-Specific Microbiome for the Advancement of RWV-Derived Tissue Models

Recent research has implicated virus-host-microbiome interactions in the processes of viral entry, pathogenesis and the host immune response [91,92,93,94,95,96]. For example, in the FRT, commensal microbiota play a key role in maintaining a healthy, homeostatic microenvironment, and disruption of this microbiota increases risk for STI acquisition. Our laboratory has successfully modeled commensal and bacterial vaginosis-associated bacteria from the vaginal microbiome using 3D RWV-derived vaginal aggregates [97,98]. Virus-microbiome interactions can also be studied using other mucosal models (e.g., lung and intestinal models), and site-specific microbiota should be included in future tissue modeling of mucosal sites [97]. Use of clinical samples or isolates may be required to recapitulate the microbial milieu at these sites. Culturing of 3D aggregates with site-specific microbiota in the context of viral infection could provide novel insights into virus-host and virus-microbiome mechanisms.

### 9.2. Advancing RWV Bioreactor Tissue Models with Enhanced Cellular Complexity

Human tissues are complex multi-cellular microenvironments, and interactions between different cell types in a tissue will influence host immune mechanisms. Co-culturing with lymphocytes and other immune cells increases the complexity of the tissues that can be modeled in the RWV bioreactor and can more faithfully recapitulate the host immune response; however, it is challenging to create autologous environments with the limited availability and lifespan of primary cell lines and optimization of diverse culture requirements. The application of stem cells and stromal cells to the RWV bioreactor may further enhance the tissue complexity of the 3D aggregates. For example, neural stem cells and neural progenitor cells are able to differentiate and develop into neurons and glia when grown in the RWV bioreactor [99,100,101]. Other examples include embryonic stem cells, pluripotent stem cells and mesenchymal stem cells, all which have been successfully propagated in RWV bioreactors and could advance RWV bioreactor-derived models [100,102,103,104,105]. The increasing utilization of RWV bioreactors for cell lines from a wide variety of tissues holds the promise of enhancing our ability to study emerging viruses that have drastic public health implications. New therapeutic agents and antiviral drugs can also be screened for toxicity and efficacy using 3D human aggregates to give a more faithful representation of in vivo microenvironments and provide superior preclinical data prior to advancing to clinical trials. Although challenging, advancing RWV bioreactor model systems to reproduce these complex in vivo cell-cell interactions could improve the translational impact of these robust 3D models in elucidating key virus-host interactions.

## 10. Conclusions

In this review, we have highlighted studies using 3D RWV-derived models to investigate virus-host interactions (Table 1). Three-dimensional RWV bioreactor-derived aggregates express cellular architectural and structural features not readily expressed in conventional monolayer cell culture, including tight junction proteins, mucus and microvilli, that are important for the analysis of virus-host interactions. Fully-differentiated aggregates can be further utilized in highly reproducible downstream gene expression analyses, high throughput and “omics” analyses, microscopy, toxicology/drug development and gene editing studies. In conclusion, RWV-derived 3D models can be employed to better understand the key interactions that influence viral pathogenesis and the host immune response to viral infection in a physiologically-meaningful context that enhances their translational utility.

## Figures and Tables

**Table 1:** Host-virus interactions in RWV bioreactor-derived 3D aggregates.

Tissue Model	Cell Lines	Virus	Virus Replication	Host Response	Reference
Neuronal	NHNP	VZV	Productive infection	No CPE	[31]
Tonsil	Primary cells	HIV	Productive infection	Lymphocyte migration tracked	[32]
Lymphoid	P3HR-1	EBV	Suppression of EBV reactivation	ND	[33]
	P3HR-1 Daudi Ramos	EBV	Suppression of EBV reactivation	ND	[34]
	BJAB Raji	EBV	Suppression of EBV reactivation	Microgravity and radiation increased DNA damage in EBV positive cells	[35]
Lung	HBTC BEAS-2B	SARS-CoV	No SARS-CoV replication detected	Vacuolization, mitochondria loss and chromatin alterations	[36]
	HBTC BEAS-2B	RSV	Productive infection	Signs of cellular damage; mucus produced in 3D aggregates	[37]
	HBTC BEAS-2B	RSV PIV3	Productive infection	Cytokine profile in 3D aggregates was similar to human airways from RSV- and PIV3-infected patients	[30]
Liver	Huh7	HCV	Productive infection	Expression and localization of TJ proteins enhance HCV infection	[38]
	PLC/PRF/5	HEV	Productive infection	No CPE	[39]
Small Intestine	INT-407	HuNoV	Increase in viral RNA copies detected	Vacuolization, shortening of apical microvilli, cell detachment from bead	[40]
			No HuNoV replication detected	Clumping and detachment of cells from microcarrier beads	[41]
			No HuNoV replication detected	No CPE	[42,43]
Colon	Caco-2	HuNoV	Increase in HuNoV RNA copies detected	Shortening of apical microvilli and formation of vacuoles	[44]
			No HuNoV replication detected	No CPE	[42,43]
		CVB	Productive infection	Increased expression of proliferation and differentiation genes	[24]
Placenta	TBPC	HCMV	Productive infection	ND	[45]
	JEG-3	VSV	No VSV replication detected	Resistance to infection mimics in vivo response to infection	[26]
Vagina	V19I	HSV-1 HSV-2	Productive infection	Mucosal epithelial barrier features mimic in vivo characteristics, aggregates are more resistant to HSV infection	[28]

NHNP, normal human neural progenitor; VZV, Varicella zoster virus; HIV, human immunodeficiency virus; HCV, hepatitis C virus; EBV, Epstein–Barr virus; HEV, hepatitis E virus; CBV, Coxsackie B virus; HCMV, human cytomegalovirus; VSV, Vesicular stomatitis virus; HSV, herpes simplex virus; RSV, respiratory syncytial virus; PIV3, parainfluenza virus type 3; SARS- CoV, severe acute respiratory syndrome coronavirus; HuNoV, human norovirus; CPE, cytopathic effects; ND, not determined.
